# Fluid Overload in Children Following Hematopoietic Cell Transplant: A Comprehensive Review

**DOI:** 10.3390/jcm13216348

**Published:** 2024-10-23

**Authors:** Lama Elbahlawan, Amr Qudeimat, Ray Morrison, Alexandra Schaller

**Affiliations:** 1 Division of Critical Care Medicine, St. Jude Children’s Research Hospital, Memphis, TN 38105, USA; ray.morrison@stjude.org; 2 Department of Bone Marrow Transplantation and Cellular Therapy, St. Jude Children’s Research Hospital, Memphis, TN 38105, USA; amr.qudeimat@stjude.org; 3 Division of Critical Care Medicine, Le Bonheur Children’s Hospital, Memphis, TN 38103, USA; alexandra.schaller@stjude.org

**Keywords:** fluid overload, fluid accumulation, hematopoietic cell transplant, pediatrics, critically ill, outcome, acute kidney injury

## Abstract

Fluid overload significantly increases morbidity and mortality in critically ill children. Following hematopoietic cell transplant (HCT), children are at a high risk of fluid accumulation due to essential increased fluid intake for nutrition, blood products, and antimicrobials. In addition, many complications predispose these children to capillary leak and fluid overload (FO), such as sinusoidal obstruction syndrome, engraftment syndrome, sepsis, and acute kidney injury (AKI). FO > 10% occurs in nearly half of children following HCT and is associated with a lower PICU survival rate. In addition, in children with acute respiratory failure post HCT, each 1% increase in cumulative fluid balance on d 3 increases the odds of PICU mortality by 3%. Furthermore, FO worsens AKI. Tools such as the renal angina index and urinary biomarkers such as neutrophil gelatinase-associated lipocalin can help identify patients at risk of AKI and FO. Early detection, prevention, and intervention are crucial to improving outcomes in this population. Management strategies include fluid restriction, diuretics, and continuous kidney replacement therapy (CKRT) when FO exceeds 10% and other measures have failed.

## 1. Introduction

Hematopoietic cell transplant (HCT) is increasingly utilized as a curative treatment for malignant and non-malignant diseases. However, the rates of morbidity and mortality following HCT are high, not just due to disease relapse but also secondary to complications arising from the treatment itself. Recently, fluid overload (FO) has been identified as a significant risk factor that increases mortality in critically ill children. Children post HCT often develop FO due to the significant volume of infusions from total parental nutrition (TPN), antimicrobial agents, immunosuppressive drugs, and blood product transfusions. In addition, complications following HCT contribute to fluid retention and overload. Cytokine release syndrome, as encountered in sepsis or engraftment syndrome, can lead to capillary leak and edema. In addition, other complications such as sinusoidal obstruction syndrome (SOS) and acute kidney injury (AKI) increase the risk of FO. In addition to its contribution to a higher risk of mortality, FO can worsen AKI and acute respiratory failure (ARF). This review highlights the effect of FO on outcomes in critically ill children following HCT and explores the impact of FO on common pathologic conditions, such as ARF, AKI, and sepsis, in this population.

## 2. Fluid Overload Pathophysiology

Fluid management in critical care medicine is fundamental to patient care. Not unlike the use of vaccines in the 1900s, the appropriate use of fluids is pivotal to changing current outcomes in ICU populations. The dogma behind fluid management has evolved, with a focus on using fluid as a medication providing therapeutic benefit but realizing its potential adverse side effects. Conservative fluid use, closer monitoring of fluid status, and detailed attention to the physiologic effects of resuscitation have become the gold standard. The interplay of fluid overload within the body is multifaceted. When the volume of fluid in is greater than the volume of fluid out, the accumulation leads to fluid deposition in extracellular compartments. Eventually, the positive fluid status leads to fluid congestion and increased cardiac filling pressure, leading to clinical signs of fluid overload and end-organ dysfunction [1,2]. Thus, while initial resuscitation may be needed, fluid may quickly go from being a supportive agent to negatively affecting the clinical status. Understanding where a patient’s fluid status sits on the spectrum can often be unclear and continuously changing. Fluid can be beneficial in restoring perfusion and end-organ function, but it can quickly play a role in capillary leak, disruption of cell-to-cell interactions, metabolic diffusion, and, ultimately, end-organ dysfunction [1]. Using appropriate assessment techniques allows for rapid assessment and intervention. Fluid balance should be evaluated frequently and closely to understand each individual patient’s fluid evolution throughout treatment. A well-rounded assessment includes clinical examination and history, diagnostic testing to observe end-organ function, and an evaluation of biomarkers.

## 3. Fluid Overload Definition

FO/cumulative fluid accumulation is calculated mainly by two methods: fluid intake/fluid output or a weight-based method.

The formula using fluid balance is:

Fluid input − fluid output (L)/weight (kg) × 100

The formula using weight is:

Current weight − admission weight (kg)/weight (kg) × 100

The most used weight in the denominator is the ICU admission weight, but other weights, such as the hospital admission weight or baseline weight, have been used in some studies.

## 4. Fluid Overload Assessment

**Figure 1:**
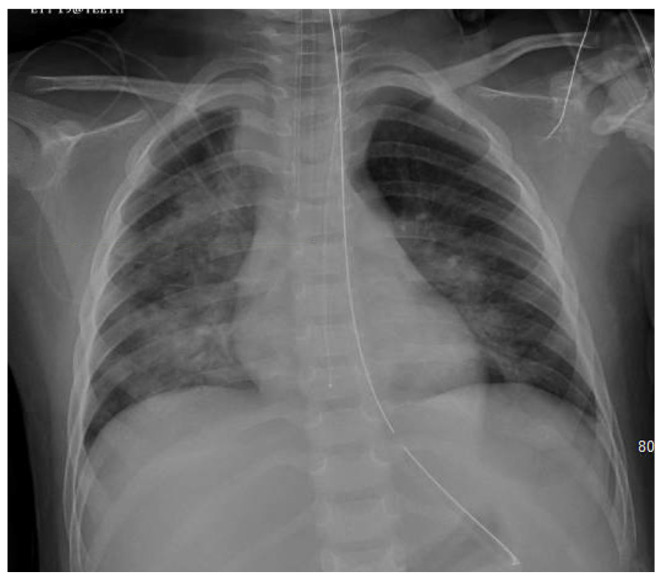
AP portable chest X-ray of a 6-year-old boy with acute respiratory failure and fluid overload showing bilateral perihilar patchy opacities.

History and physical examinations should be used to understand the patient’s status. Table 1 outlines the diagnostic methods and findings of fluid overload. Chest radiography is easily accessible and offers an initial insight into a patient’s fluid status. X-rays can show signs of volume overload, such as cardiomegaly, interstitial edema, dilated pulmonary artery, pleural effusions, alveolar edema, or Kerly lines (Figure 1) [1]. Although radiography is a good tool for initial evaluation and insight, studies have shown that chest radiographs lack both sensitivity and specificity. The technique, status of the patient, positioning, and use of portable X-rays can decrease this sensitivity even further [1].

### 4.1. Ultrasound

**Figure 2:**
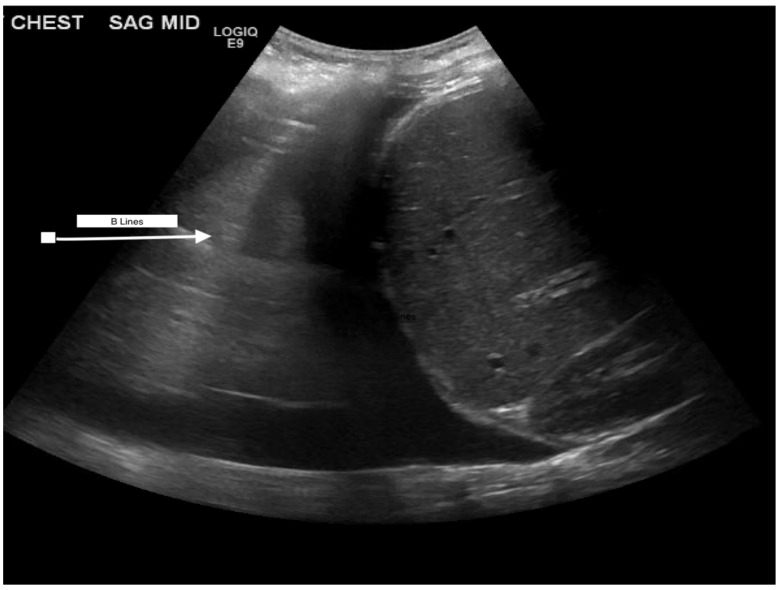
B-lines in thoracic ultrasound in a child with pulmonary edema. The ultrasound appearance is of a vertical, discrete, hyperechogenic line that arises from the pleural line and extends to the bottom of the screen, moving synchronously with respiration.

Ultrasound offers a non-invasive and consequential look into fluid overload. Basic evaluation for FO on a cardiac ultrasound may include left ventricular ejection fraction based on wall mobility and the size of the ventricle, the presence of a pericardial effusion, or clear valvular dysfunction [2]. More advanced investigations of FO using cardiac ultrasound can include right ventricular enlargement with intraventricular septum flattening in only diastole, as well as tricuspid regurgitation secondary to right ventricular enlargement. Furthermore, fluid responsiveness can be tested via the monitoring of stroke volume and LV outflow tract velocities by using a Doppler [2]. In addition, inferior vena cava (IVC) size and collapsibility can add to the clinical assessment of fluid status and give a basic estimate of right atrial pressure. An IVC size of <1.5 cm signifies volume depletion status, but a size of >2.5 cm suggests volume overload. In patients with appropriate fluid status, the IVC size will be between 1.5 and 2.5 cm, and the diameter will vary with inspiration and expiration [1]. In addition, ultrasound can easily identify edema within the lung tissue by demonstrating A- and B-lines when interstitial thickening is present from, most notably, fluid. Increased B-lines correlate with increased pulmonary edema (Figure 2) [2]. Clinical assessment must be used in conjunction with lung ultrasound findings because interstitial thickening can be due to factors other than fluid. Ultrasound can show other signs of increased fluid with bowel wall edema, ascites, and jugular vein pressure [2,3]. A novel technique introduced in cardiac patients quantifies increased right atrial pressures, as seen in fluid overload, by looking at downstream effects on organs. The protocol assesses systemic venous congestion by examining Doppler flows in the hepatic, portal, and renal arteries. Venous excess ultrasound (VExUS) grading helps to understand abnormalities of flow in these veins. High abnormalities in Doppler flow indicate organ congestion. In conjunction with a dilated IVC, a high risk of AKI could be predicted [2].

### 4.2. Bioimpedance Analysis

**Figure 3:**
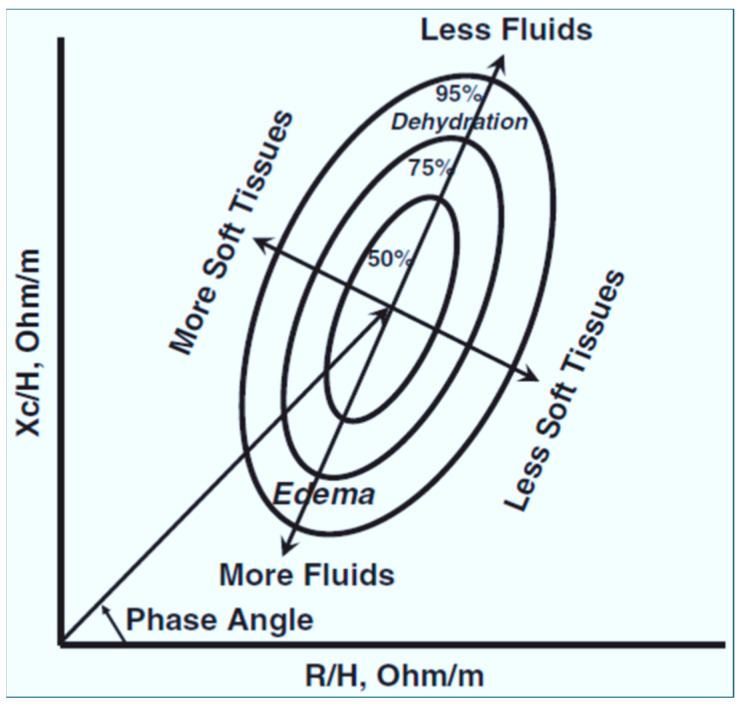
Bioelectrical impedance vector analysis (BIVA) enables classification of under-, normal, and over-hydration.

Although not commonly used in PICUs, bioimpedance analysis examines alternating currents to measure body composition, including soft-tissue hydration [1,2]. This tool uses body impedance and resistance to estimate fluid status (Figure 3) [4]. Further studies are required to understand how applicable and useful this technique is in the critical care setting, but it has shown a correlation with central venous pressure and may offer insight into fluid status on admission [1].

### 4.3. Biomarkers

Biomarkers used in conjunction with other tools may help provide insight into fluid status and the status of underlying illness. B-type natriuretic peptide (BNP) has shown excellent negative predictive value in the diagnosis of heart failure and shows down-trending in the setting of diuretic therapy for heart failure and cardiomyopathy but still requires further studies to drive therapy [2]. NT-proBNP, a precursor of BNP, can also be used as a marker for fluid overload. NT-proBNP has a longer half-life, making it less vulnerable to acute changes that affect BNP. Studies have also shown that NT-BNP is superior in predicting morbidity and mortality in the setting of heart failure [5]. Other biomarkers, such as lactate, hemoglobin, and hematocrit, are less specific to fluid overload but may provide insight into the adequacy of end-organ perfusion and fluid effects such as hemodilution [2].

Individually, all these tests give insight into fluid status, but likely, a combination of many of these tests and the creation of predictive tests will lead to optimal clinical decision making. An example of this is the recently reported Endothelial Activation and Stress Index (EASIX) [6]. In states of hypervolemia, adult patients following HCT have increased amounts of endothelial glycocalyx layer, a part of the capillary permeability barrier, as well as increased atrial natriuretic peptide. EASIX, a marker of endothelial dysfunction, is defined as lactate dehydrogenase (U/L) × creatinine (mg/dL)/platelets (10^9^ cells/L]. In adults post HCT, an EASIX score of >4.4 at admission in the study cohort (145 patients, haploidentical) was a significant predictor of grade ≥2 FO (hazard ratio (HR) = 4.8, *p* < 0.001) [6]. Likewise, a high EASIX score on admission was associated with FO grade ≥ 2 in the validation cohort (449 patients, HLA matched), with a cutoff value of 4.3 (HR = 4.8, *p* < 0.001). Future studies are needed to validate the association of this index with FO in the post HCT pediatric population.

## 5. Fluid Overload in the Transplant World

FO is highly prevalent in children during their post HCT course. In a cohort of 484 children post HCT, FO > 10% occurred in 46% of the total cohort and in 67% of those with severe AKI [7]. In addition, FO is a strong predictor for PICU admission in these children [8]. Benoit et al. followed up 87 children who underwent HCT and compared their maximal weight gain (WG) and cumulative fluid balance from the day of admission for transplant to those on the day of ICU admission (PICU group, 19 patients) or hospital discharge (non-PICU group, 68). WG > 10% was strongly associated with PICU admission and was encountered in 68.4% of patients in the PCU group and 22.1% of patients in the non-PICU group [8]. Recently, Rondon et al. proposed the following grading system for fluid toxicity in patients post HCT: grade 1, WG < 10% from baseline—might require decreasing IV fluids or occasional diuretics; grade 2, symptomatic and/or WG 10 to <20% from baseline—might require continuing diuretics; grade 3, WG ≥ 20% from baseline or not responding to diuretics; and grade 4, major organ dysfunction (renal, pulmonary, or cardiac dysfunction) or requiring intensive care [9]. In their cohort of 145 adults post haploidentical HCT, FO grade ≥ 2 was an independent predictor of D +100 non-relapse-related mortality (HR = 13, *p* < 0.001) [9]. Table 2 summarizes the results of the published studies on FO in children following HCT.

## 6. Fluid Overload in the Critical Care World

Similarly, FO is often encountered in critically ill children and is associated with poor outcomes. A large meta-analysis (44 studies, 7507 children) reported a pooled median FO incidence of 33% in critically ill children (range, 10–83%) [3]. A recent meta-analysis of 44,682 critically ill children from 120 studies confirmed that fluid accumulation (FA) during PICU admission was strongly associated with mortality [17]. Each 1% increase in the percentage of FA was associated with 6% increased odds of mortality. Importantly, FA within 24 h of PICU admission increased the risk of mortality significantly, with an OR of 7.93 (95% CI, 2.81–22.39; *p* < 0.001) for FA > 5% and an OR of 8.77 (95% CI, 2.42–31.77; *p* < 0.001) for FA > 10%. This is particularly important considering that almost half of children post HCT experience FO > 10% [7]. Therefore, the early detection and management of FO in these children is crucial in the transplant unit and in the early stage of PICU admission.

## 7. Fluid Overload and Acute Kidney Injury

**Figure 4:**
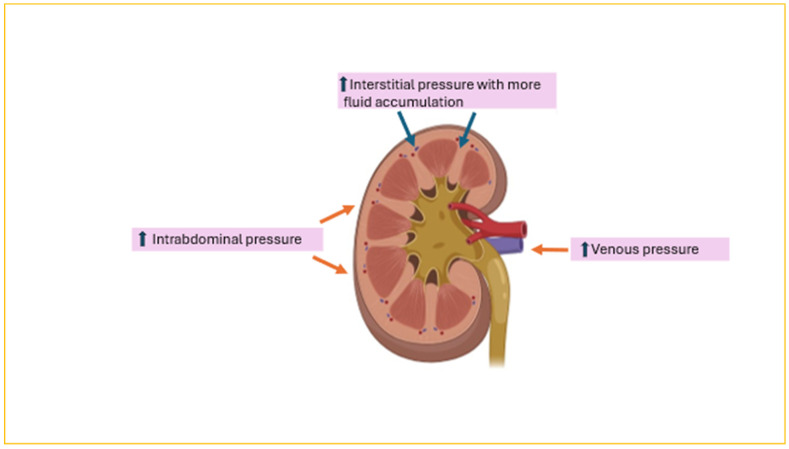
Impact of fluid overload on the kidney.

AKI is common and encountered in half of children following HCT, with severe AKI occurring in 12% [18,19]. Most importantly, AKI reduces the 3-year overall survival rate from 79.6% in children with no AKI to 46.7% in children with stage 2 AKI and 25% in those with stage 3 AKI (*p* = 0.025 and 0.002, respectively) [20]. In a pediatric cohort of 484 post HCT patients, AKI developed in 38%, 42% of whom experienced severe AKI [7]. Moreover, FO > 10% was present in 67% of children with severe AKI post HCT. The risk of death was significantly higher in patients with severe AKI than in those without AKI (HR, 4.6). The relationship between FO and AKI is complex (Figure 4). FO can exacerbate kidney injury by worsening kidney venous hypertension from increased central venous pressure, impairing perfusion pressure capacity of the glomerular capillaries, or raised intra-abdominal pressure. In addition, the kidney is an encapsulated organ; thus, FO will raise interstitial pressure, leading to compromised blood flow. Conversely, AKI itself can exacerbate FO secondary to the renal impairment itself. As a result, a vicious cycle is perpetuated whereby each condition exacerbates the other. The largest meta-analysis to date (44,682 critically ill children) confirmed an increased risk of AKI (OR = 1.98 [95% CI, 1.60–2.44]; *p* < 0.001) with fluid accumulation. Balancing the management of FO without exacerbating AKI, especially in the context of diminished intravascular volume from capillary leak or acute tubular necrosis due to renal hypoperfusion, is quite challenging. Fluid restriction and diuretics are usually the first lines of management. Subsequently, continuous kidney replacement therapy (CKRT) is initiated if these measures fail to improve FO. However, a more systemic proactive approach to FO detection and management at the time of AKI, such as using the renal angina index (RAI) and urinary biomarkers such as neutrophil gelatinase-associated lipocalin (uNGAL), could reduce mortality. The RAI combines risk stratification and clinical signs of injury [21]. Risk stratification elements include ICU admission, stem cell transplantation, and mechanical ventilation or inotropic support. Clinical signs of injury are based on changes in estimated creatinine clearance or the percentage of fluid overload (%FO). An RAI score of ≥8 indicates a higher risk of occurrence of AKI by day 3 of PICU admission [22]. Goldstein et al. tested the use of automated clinical decisions in 286 critically ill children using the RAI and uNGAL levels [23]. This approach resulted in a reduced rate of FA ≥ 15% prior to CKRT and an 11-day shorter length of stay in the ICU as well as higher survival rates to ICU discharge.

## 8. Fluid Overload and Acute Respiratory Failure

ARF is a serious complication seen in 25% of children post HCT, with a high mortality rate of 42–64%% [24,25]. FO has been implicated in previous studies associated with higher mortality rates, fewer ventilator-free days, and oxygen deficits in critically ill children. The pooled data from 12 pediatric studies with 1819 patients confirmed the association of FO with a prolonged duration of mechanical ventilation (weighted mean difference = 38.1 h [95% CI, 19.35–56.84]; *p* < 0.001) [17]. FO also affects outcomes in children with ARF post HCT. In a cohort of 198 post HCT intubated children with ARF, the cumulative fluid balance on d 3 of their invasive mechanical ventilation (IMV) course was significantly associated with mortality [16]. Each 1% increase in cumulative fluid balance (CFB) on d 3 increased the odds of PICU mortality by 3% (OR 1.03, 95% CI, 1.00–1.07). In addition, higher CFB on d 3 was associated with a lower rate of extubation at d 28 and d 60 [16]. In another cohort of 30 intubated post HCT children with acute respiratory failure secondary to engraftment syndrome, lower median cumulative FO% on d 4 and d 5 after the start of IMV was observed in survivors (2.8 vs. 14.0 mL/kg, *p* = 0.038 on day 4 and 1.8 vs. 14.9 mL/kg, *p* = 0.044 on day 5, respectively) [14]. Similar findings are reported in critically ill children with pediatric acute respiratory distress syndrome. In a large cohort of 723 children with ARDS, a positive CFB on days 5 through 7 was associated with a higher mortality rate [26]. In addition, a higher CFB on days 4 to 7 was associated with a lower probability of successful extubation.

## 9. Fluid Overload and Continuous Kidney Replacement Therapy

CKRT is used to manage FO when other measures, such as fluid restriction and diuretics, have failed. FO is the main indication for initiation of CKRT in children post HCT [11]. In a large meta-analysis that examined the effect of FA in critically ill children, FA at the time of initiation of kidney replacement therapy (KRT) was associated with a higher mortality rate. In children with FA% > 10% and FA > 20%, the pooled OR for mortality was 2.96 (95% CI, 1.85–4.73; *p* < 0.001; I 2 = 80%; *n* = 2488) and 2.91 (95% CI, 1.82–4.63; *p* < 0.001; I 2 = 68%; *n* = 1991), respectively [17]. The same deleterious effect of FO at the time of initiation of CKRT was observed in a pediatric oncology cohort (total number of patients = 68, post HCT = 23) from eight PICUs in the Netherlands [13]. Children with FO > 10% at CKRT initiation were 6.16 times more likely to die than were those with FO ≤ 10%. Thus, the timing of initiation of CKRT is critical and should be considered before FA exceeds 10%. Unfortunately, the mortality of children post HCT receiving CKRT continues to be high (52–65%). Risk factors other than FO that are associated with the higher mortality rate include vasoactive support and mechanical ventilation [13,27]. The management of FO with CKRT typically involves a slow and consistent rate of net fluid removal over an extended period. This approach makes CKRT more tolerable from a hemodynamic standpoint. Aggressive and fast fluid removal during CKRT may affect not only blood pressure but also survival. Studies of adults suggest that higher net ultrafiltration rates (>1.75 mL/kg/h) increase the risk of mortality and reduce the probability of kidney recovery in patients receiving CKRT [28,29].

## 10. Fluid Overload and Sepsis

Sepsis is associated with a higher risk of mortality in children post HCT, particularly increasing the risk of hospital mortality to four-fold that of septic children with no history of HCT [30]. In the early phase of septic shock, fluid resuscitation is key to restoring adequate tissue perfusion. The Surviving Sepsis Campaign guidelines published in 2021 recommend that 30 mL/kg of IV crystalloid fluid be given within the first 3 h of resuscitation [31]. Additional fluid needs should be guided by the patient’s hemodynamic status and responsiveness to fluid boluses. The Surviving Sepsis Campaign guidelines did not have recommendations for either restrictive or liberal fluid management in the first 24 h of resuscitation after the initial fluid bolus. Furthermore, in the FEAST Trial, which included 3141 children with sepsis in Africa, fluid boluses significantly increased the 48 h mortality rate in critically ill children with impaired perfusion [32]. Therefore, it is prudent to continuously evaluate the patient’s status and fluid responsiveness, as fluid administration may become more harmful beyond the initial state of resuscitation.

## 11. Other Transplant-Related Complications and Fluid Overload

Hepatic sinusoidal obstruction syndrome (SOS), also known as veno-occlusive disease of the liver, is a condition that is well known to be associated with FO. The pathophysiology stems from prep regimen-related injury to the sinusoidal endothelium of the liver, ultimately leading to the embolization of the centrilobular vein and subsequent post-sinusoidal obstruction [33,34]. This, in turn, leads to the classic presentation of ascites, weight gain, abdominal pain, and jaundice. Furthermore, FO is exacerbated by accompanying acute renal injury and frequent platelet transfusions triggered by SOS-related consumptive thrombocytopenia. Fluid accumulation in SOS is extravascular; thus, diuresis can only partially correct fluid status. Defibrotide, approved by the FDA in 2016 for the treatment of SOS, promotes endothelial cell proliferation and the partial revascularization of the sinusoid, leading to lower mortality rates than achieved by supportive treatment only [33,34]. Despite treatment with defibrotide, most patients still require additional supportive measures, such as fluid restriction and diuretic therapy, and some will have progressive acute renal injury, with poor urine output requiring renal replacement therapy.

The constellation of fever, rash, and weight gain around the time of neutrophil recovery in the early post HCT period characterizes engraftment syndrome. This could be accompanied by any combination of liver dysfunction, renal dysfunction, or encephalopathy [35]. The release of cytokines, including IL-1, TNF-alpha, and IFN-gamma, during engraftment leads to capillary leak and third space fluid loss that is responsible for the weight-related, pulmonary, and renal components of engraftment syndrome. Similar to SOS, the intravascular volume status is not repleted, making it difficult to correct the fluid balance state with diuretics alone. Although engraftment syndrome can be self-limited in some patients, requiring no therapy, patients with severe disease require intervention. This includes patients with high fevers and oxygen requirements due to pulmonary edema. These patients may be started on intravenous methyl prednisone at 1 mg/kg/day and weaned over a few days according to response. Diuresis can also be considered to aid fluid and weight management.

## 12. Management

The prevention of FO is the cornerstone of management. In children following HCT, FO prevention should start on the ward before PICU admission. Initially, on PICU admission, fluid may be needed to restore end-organ perfusion, but it is crucial to reduce fluid administration once it starts to become detrimental. Even maintenance fluids can contribute to gradual fluid accumulation due to “fluid creep”. Fluid creep is the sum of the volumes of electrolytes, the small volumes to keep venous lines open (saline or glucose 5%), and the total volume used as a vehicle for medication and accounts for nearly one-third of the mean daily fluid volume [36,37]. Therefore, to avoid reducing nutritional intake, it is important to minimize the volume of fluid creep. The ReLiSCh Trial compared restrictive fluid therapy (40% of maintenance fluids) with usual/liberal fluid therapy (70–80% of maintenance fluids) in mechanically ventilated children. The restricted group had significantly more ventilator-free days (23 vs. 17 d, *p* = 0.008) and PICU-free days (19 vs. 15 d, *p* = 0.007). These findings are comparable to those in the earlier FACTT trial, which included 1000 adult patients with acute lung injury. A restrictive fluid strategy was associated with a shorter duration of mechanical ventilation and ICU length of stay but not with decreased mortality [38]. Although the debate about whether to use restrictive or non-restrictive fluid administration continues, a proactive approach to prevent FO is crucial. This includes accurately monitoring daily fluid intake and output, restricting volume (particularly fluid creep), and administering intermittent or continuous infusion of diuretics as needed. CKRT should be considered if FO approaches 10% despite other treatments.

## 13. Conclusions

In conclusion, reducing complications after HCT enhances the success of this therapy and improves survival. The prompt detection and treatment of fluid accumulation should be fundamental in caring for these children upon hospitalization or PICU admission. FO not only raises the risk of mortality but also exacerbates the severity and likelihood of other complications, such as AKI and ARF. Hospitals should implement a systematic approach for early recognition and management, including electronic health record alerts, to ensure early detection.

## Figures and Tables

**Table 1:** Diagnostic evaluation of fluid overload.

Diagnostic	Findings of FO	Other Findings	Pros/Cons
CXR	Cardiomegaly, interstitial edema, dilated pulmonary artery, pleural effusion, alveolar edema, Kerly lines		Easily obtained/good initial screening Lacks sensitivity and specificity
Ultrasound			
Cardiac	Presence of pericardial effusion, poor LV ejection fraction, valvular dysfunction, right ventricular enlargement, intraventricular septum flattening in diastole, tricuspid regurgitation, LV outflow tract velocities with Doppler		Easily obtained/good initial screening Similar findings can be present for different etiologies
	IVC collapsibility	Diameter <1.5 cm = volume depleted status >2.5 cm = volume overload w/no variability with breathing	Lacks sensitivity and specificity
Abdominal	Evaluate hepatic vein, portal vein, intrarenal artery for end organ perfusion due to increased right atrial pressure, bowel wall edema, ascites		Easily obtained, lacks sensitivity and specificity
	Venous congestion with Doppler	Diameter > 2 cm indicates congestion in IVC Edema is notable if B-lines present	Requires more evidence
Bioimpedance Analysis	Alternating currents to measure body composition and soft-tissue hydration		Not easily accessible Requires more evidence
Biomarker			
BNP	Secreted by cardiomyocytes under stretch condition	Down trending during diuretic therapy in patients with heart failure	Excellent negative predictive value in diagnosis of heart failure
Urinary NGAL	Increased early in AKI		Reduced rate of FO ≥ 15% prior to CKRT and a shorter length of stay in PICU when combined with RAI

FO—fluid overload; IVC—inferior vena cava; BNP—B-type natriuretic peptide; NGAL—neutrophil gelatinase-associated lipocalin; AKI—acute kidney injury; CKRT—continuous kidney replacement therapy; PICU—pediatric intensive care unit; RAI—renal angina index.

**Table 2:** Summary of fluid overload studies in children following hematopoietic stem cell transplant.

Author, Year	Population (n)	Fluid Balance	Outcome	Main Findings
Michael et al., 2004 [10]	Allogeneic (26) Acute renal failure Admitted to PICU (23)	Cumulative fluid input and output	Survival Euvolemia (FO < 10%)	Survival rate was 42% Survival rate in children who received KRT was 29% All survivors were euvolemic
Benoit et al., 2007 [8]	Allogeneic and Auto (87) HCT to PICU admission (19) HCT to hospital discharge (68)	Weight-based Cumulative fluid input and output	PICU admission	Weight gain >10% on admission to HCT is a risk factor for PICU admission
Flores et al., 2008 [11]	Stem cell (51) CKRT (51)	Cumulative fluid input and output	PICU survival	FO was the most common reason for CRRT initiation Survival to PICU discharge was 45%
Lombel et al., 2012 [12]	Stem cell (21) CKRT (21)	Weight-based Cumulative fluid input and output	PICU mortality PELOD scores	Survival to PICU discharge was 61% FO% was not associated with PICU mortality FO was predictive of higher subsequent PELOD scores
Raymakers-Janssen et al., 2019 [13]	Cancer and HCT (68) HCT (23) CKRT (68)	Weight-based	PICU mortality	Survival to PICU discharge was 45.6% Survival of HCT patients to PICU discharge was 39.1% FO and inotropic support at the start of CRRT were associated with PICU mortality
Elbahlawan et al., 2020 [14]	Allogeneic and Auto (30) ARF/IMV (30)	Cumulative fluid input and output	PICU survival	Survival to PICU discharge was 80% Lower cumulative FO% in survivors on D4 and D5 after start of IMV
Mueller et al., 2020 [15]	Allogeneic and Auto (92)	Cumulative fluid input and output	PICU mortality	Fluid balance 24 h prior and 24 h post PICU admission was not associated with survival, ventilator-free days, or PICU-free days
Sallee et al., 2021 [16]	Allogeneic (198) ARF/IMV (198)	Cumulative fluid input and output	Effect of cumulative fluid balance on outcome	Positive D3 CFB was associated with higher PICU mortality and a lower rate of extubation
Bauer et al., 2023 [7]	Allogeneic and Autologous (484) AKI (168)		AKI	FO > 10% was present in 67% of patients with severe AKI Risk of death at 1 year was higher in severe AKI than in those with no AKI (HR, 4.6)

HCT—hematopoietic cell transplant; PICU—pediatric intensive care unit; KRT—kidney replacement therapy; CKRT—continuous kidney replacement therapy; AKI—acute kidney injury; ARF—acute respiratory failure; IMV—invasive mechanical ventilation; CFB—cumulative fluid balance; FO—fluid overload; PELOD—PEdiatric Logistic Organ Dysfunction.

## Data Availability

No new data were created or analyzed in this study. Data sharing is not applicable to this article.
